# BMS-986020, a Specific LPA_1_ Antagonist, Provides Neuroprotection against Ischemic Stroke in Mice

**DOI:** 10.3390/antiox9111097

**Published:** 2020-11-08

**Authors:** Bhakta Prasad Gaire, Arjun Sapkota, Ji Woong Choi

**Affiliations:** Laboratory of Neuropharmacology, College of Pharmacy and Gachon Institute of Pharmaceutical Sciences, Gachon University, Incheon 21936, Korea; samarpanbp@gmail.com (B.P.G.); sapkotaa07@gmail.com (A.S.)

**Keywords:** BMS-986020, transient middle cerebral artery occlusion (tMCAO), neuroprotective effects, long-term neuroprotection, neurogenesis, angiogenesis

## Abstract

Stroke is a leading cause of death. Stroke survivors often suffer from long-term functional disability. This study demonstrated neuroprotective effects of BMS-986020 (BMS), a selective lysophosphatidic acid receptor 1 (LPA_1_) antagonist under clinical trials for lung fibrosis and psoriasis, against both acute and sub-acute injuries after ischemic stroke by employing a mouse model with transient middle cerebral artery occlusion (tMCAO). BMS administration immediately after reperfusion significantly attenuated acute brain injuries including brain infarction, neurological deficits, and cell apoptosis at day 1 after tMCAO. Neuroprotective effects of BMS were preserved even when administered at 3 h after reperfusion. Neuroprotection by BMS against acute injuries was associated with attenuation of microglial activation and lipid peroxidation in post-ischemic brains. Notably, repeated BMS administration daily for 14 days after tMCAO exerted long-term neuroprotection in tMCAO-challenged mice, as evidenced by significantly attenuated neurological deficits and improved survival rate. It also attenuated brain tissue loss and cell apoptosis in post-ischemic brains. Mechanistically, it significantly enhanced neurogenesis and angiogenesis in injured brains. A single administration of BMS provided similar long-term neuroprotection except survival rate. Collectively, BMS provided neuroprotection against both acute and sub-acute injuries of ischemic stroke, indicating that BMS might be an appealing therapeutic agent to treat ischemic stroke.

## 1. Introduction

Receptor-mediated lysophosphatidic acid (LPA) signaling influences diverse biological processes [1,2]. LPA receptors have been suggested as promising targets for drug development to treat various diseases by employing experimental disease models [1,2,3]. These preclinical efforts have been closer achieving clinical success because of BMS-986020 (BMS). BMS, also known as AM152, is a selective antagonist for LPA receptor 1 (LPA_1_) [4,5,6,7]. It has been reported to have an IC_50_ value < 300 nM for human LPA_1_, whereas that for LPA_3_ was about 1–10 μM [6]. It has emerged as a potential drug candidate to treat fibrosis based on preclinical efficacy [8,9] and clinical possibility [10,11]. In particular, it has been demonstrated to be effective for treating idiopathic pulmonary fibrosis in phase II clinical trials (ClinicalTrials.gov Identifier: NCT01766817). It is also under another phase I clinical trial for psoriasis (ClinicalTrials.gov Identifier: NCT02763969). Considering the role of LPA_1_ in diverse experimental models, BMS might also be an effective therapeutic to treat other types of LPA_1_-associated diseases including cancer, lung injury, systemic sclerosis, neuropathic pain, spinal cord injury, hydrocephalus, hypoxia, neuropsychiatric disorders, and traumatic brain injury [12,13,14,15,16,17,18]. Besides these disease types, ischemic stroke could also be a promising target disease for therapeutic application of BMS. Stroke is caused by a sudden interruption of blood flow in the brain, resulting in lasting brain damage through diverse pathogenic mechanisms [19,20]. For stroke survivors, long-term functional disability after the stroke has been a major problem [21,22]. LPA_1_ can contribute to acute brain injuries in mice challenged with ischemic stroke by regulating neuroinflammatory responses, such as activation of microglia and upregulation of pro-inflammatory cytokines in post-ischemic brains [12]. LPA_1_ can also contribute to neuropathic pain in mice after ischemic challenge [18,23]. These independent studies strongly suggest that targeting LPA_1_ can be a considerable strategy for drug development to treat ischemic stroke. Therefore, clinically-relevant BMS might also be an effective drug candidate for ischemic stroke.

Thus, the objective of the current study was to investigate neuroprotective effects of BMS against ischemic stroke by employing a mouse model of transient middle cerebral artery occlusion (tMCAO). First, whether BMS could exert neuroprotective effects against acute brain injuries following tMCAO challenge was determined by assessing its effects on brain infarction, neurological deficits, apoptotic cell death, microglial activation, and lipid peroxidation at day 1 or day 3 after tMCAO challenge. Whether BMS administration at 3 h post-ischemic challenge could exert similar neuroprotective effects was also determined. Importantly, whether BMS could also be effective against sub-acute brain injuries following tMCAO challenge was determined by assessing its effects on neurological deficits, survival rate, and brain tissue loss up to 15 days or at the end time point after the challenge. In addition, possible mechanisms for long-term neuroprotective effects of BMS were investigated by assessing effects of BMS on neurogenesis and angiogenesis at day 15 after ischemic challenge.

## 2. Materials and Methods

### 2.1. Animals

Male ICR mice (32 ± 2 g, six weeks old) were obtained from Orient Co., Ltd. (Kyungi-do, Korea) and acclimatized at controlled laboratory conditions of diurnal lights (light on: 07:00–19:00) at humidity of 60 ± 10% and temperature of 22 ± 2 °C. All animal experiments were performed in compliance with guidelines from the Institutional Animal Care and Use at Lee Gil Ya Cancer and Diabetes Institute (LCDI) of Gachon University (No. of approved animal protocols: LCDI-2018-0051 and LCDI-2019-0027).

### 2.2. Induction of tMCAO Challenge in Mice

Focal ischemic stroke was induced through transient occlusion of right middle cerebral artery (tMCAO) in mice using an intraluminal suture occlusion method as described previously [24,25]. Briefly, mice were anesthetized with isoflurane (3% for induction and 1.5% for maintenance at ratio of 70% N_2_O:30% O_2_). Middle cerebral artery (MCA) was occluded by inserting a 9-mm-long nylon monofilament (5-0) coated with silicon from carotid bifurcation. Blood flow was restored by withdrawing the monofilament after 90 min of MCA occlusion. Sham-operated mice underwent similar surgical procedures without MCA occlusion.

### 2.3. BMS Administration

After MCA occlusion, mice were randomly assigned into a BMS- or a vehicle (1% DMSO in 10% Tween-80)-administered group. BMS was kindly provided by Dr. Dong Yun Shin (Gachon University, Incheon, Korea). To determine whether BMS could exert neuroprotective effects against acute brain injuries in tMCAO-challenged mice, BMS was administered via oral gavage at different dosages (0.5, 2, 5, and 10 mg/kg) immediately after reperfusion. For the time window experiment, BMS was orally administered at 3 h after reperfusion. To determine long-term neuroprotective effects of BMS against sub-acute brain injuries, BMS was orally administered once immediately after reperfusion for the single administration group or daily for the repeated administration group (administration for fourteen consecutive days).

### 2.4. Assessment of Neurological Deficits and Survival Rate

Neurological deficit scores were assessed using a modified neurological severity score (mNSS) at 24 h after tMCAO for short-term analysis or every day for long-term analysis. Neurological deficit scores for motor, sensory, balance, and reflex tests were assessed using an 18-point scale (0 for normal and 18 for maximum deficits), as described previously [26,27]. Besides neurological assessment, survival rate of mice subjected to experiments for long-term neuroprotection was determined by calculating the percentage of dead mice during the experimental time course.

### 2.5. Assessment of Brain Infarction

At one day after tMCAO, mice were sacrificed with CO_2_ inhalation and their brains were collected to assess brain infarction using 2,3,5-triphenyltetrazolium chloride (TTC) staining. Coronal brain sections were incubated with 2% TTC solution for 20 min at 37 °C and photographed. Obtained images were used to measure infarct volume with ImageJ (National Institute of Mental Health, Bethesda, MD, USA). Infarct area was determined by dividing damaged area by total area of each slice and summed up to obtain the total infarct area.

### 2.6. Histological Analysis

#### 2.6.1. Tissue Preparation

Brain samples for histological analyses were obtained at 1, 3, and 15 days after tMCAO challenge. Mice were anesthetized with a mixture of Zoletil 50^®^ (10 mg/kg, i.m., Virbac Laboratories, Carros, France) and Rompun^®^ (3 mg/kg, i.m., Bayer HealthCare LLC, KS, USA) and perfused with ice-cold phosphate-buffered saline (PBS) followed by fixing with 4% paraformaldehyde (PFA).

To measure brain tissue loss, harvested brains at 15 days after tMCAO challenge were photographed. The percentage of brain tissue loss in the ipsilateral hemisphere was measured using ImageJ software with the following formula: percent of brain tissue loss = (area of contralateral hemisphere − area of ipsilateral hemisphere)/area of contralateral hemisphere × 100%.

For immunohistochemistry, brains were further fixed in 4% PFA overnight and immersed in 30% sucrose solution for cryoprotection. Brains were then embedded in Tissue-Tek^®^ optimal cutting temperature compound, frozen on dry ice, and coronally sectioned (20 µm) using a cryostat (RD-2230, Roundfin, Liaoning, China).

#### 2.6.2. TUNEL Assay

To determine effects of BMS on cell apoptosis, TUNEL immunoassay was performed at 1 day and 15 days after tMCAO using an in-situ cell death detection kit (Roche, Mannheim, Germany) according to the manufacturer’s protocol. Cryostat brain sections were post-fixed in 4% PFA for 10 min and permeabilized with 0.1% sodium citrate in 0.1% Triton X-100 for 2 min on ice. Brain sections were then labelled with TUNEL assay kit for 1 h, washed with PBS, and mounted with VECTASHIELD mounting media (Vector Laboratories, Burlingame, CA, USA). Images were taken with a DP72 camera using a fluorescent microscope (BX53T, Olympus Co., Tokyo, Japan).

#### 2.6.3. Immunohistochemistry Against Iba1 or 4-HNE

To determine the effects of BMS administration on microglial activation and lipid peroxidation, immunohistochemical analysis was performed as described previously [28]. Briefly, cryostat brain sections were oxidized with 1% H_2_O_2_ for 15 min and blocked with 1% fetal bovine serum (FBS) in 0.3% Triton X-100. Sections were then labeled with a rabbit primary antibody against Iba1 (1:500, Wako Pure Chemicals, Osaka, Japan) or 4-hydroxynonenal (4-HNE, 1:500, Bioss, Freiburg, Germany) overnight at 4 °C, further labeled with an appropriate biotinylated secondary antibody (1:200, Santa Cruz Biotechnology, TX, USA), and then incubated with ABC reagent (1:100, Vector Laboratories). Brain sections were exposed to 3,3’-diaminobenzidine substrate (Dako, Santa Clara, CA, USA) to visualize Iba1- or 4-HNE-positive signals, dehydrated in ascending grade of alcohol, cleared in xylene, and mounted with an Entellan media (Merck, Darmstadt, Germany).

#### 2.6.4. Double Immunofluorescence Followed by 5-Bromo-2′-Deoxyuridine (BrdU) Incorporation

To determine effects of BMS administration on neurogenesis and angiogenesis, BrdU/DCX- and BrdU/CD31-double immunofluorescence assays were performed as described previously [12,29]. In brief, BrdU (50 mg/kg in PBS, i.p., Sigma-Aldrich, St. Louis, MO, USA) was administered to mice at 13 and 14 days after tMCAO challenge for four times at 12 h interval. For double immunofluorescence, brain sections were incubated with 2N HCl to denature DNA followed by neutralization with 0.1 M borate buffer. Sections were then blocked with 1% FBS in 0.3% Triton X-100 and simultaneously incubated—with either a rat anti-BrdU (1:400, Abcam, Cambridge, UK) and a goat anti-DCX (1:100, Santa Cruz Biotechnology) primary antibodies or a mouse anti-BrdU (1:200, ImmunoBioScience Corp., Mukilteo, Washington, DC, USA) and a rat anti-CD31 (1:300, Dianova, Hamburg, Germany) primary antibodies—overnight at 4 °C to label newly formed neurons or newly formed blood vessels. Sections were then incubated with respective secondary antibodies (1:1000) conjugated with Cy3 (Jackson ImmunoResearch, West Grove, PA, USA) or AF488 (Invitrogen) and mounted with VECTASHIELD mounting media. Images were obtained using a confocal microscope (Eclipse A1 Plus, Nikon, Japan).

#### 2.6.5. Image Preparation and Quantification

Obtained photographs were processed to prepare representative images using Adobe Photoshop Element 8 (Adobe, San Jose, CA, USA). For quantification, three different images for each mouse were obtained for brain regions. Numbers of immunopositive cells were manually counted in a blind fashion as described previously [12]. Total numbers of immunopositive cells were presented as numbers of cells per unit area.

### 2.7. Statistical Analysis

All statistical analyses were performed using GraphPad Prism 7 software (GraphPad Software Inc., La Jolla, CA, USA). Data are expressed as mean ± standard error of mean (S.E.M.). Statistical differences between two groups were analyzed using Student’s t-test and those among groups were analyzed using one-way analysis of variance (ANOVA) followed by Newman–Keuls test. Statistical difference for survival rate was analyzed by Log-rank (Mantel–Cox) test. Statistically significant difference was considered at *p* < 0.05.

## 3. Results

### 3.1. BMS Administration Attenuates tMCAO-Induced Brain Infarction and Neurological Deficits during the Acute Phase

**Figure 1:**
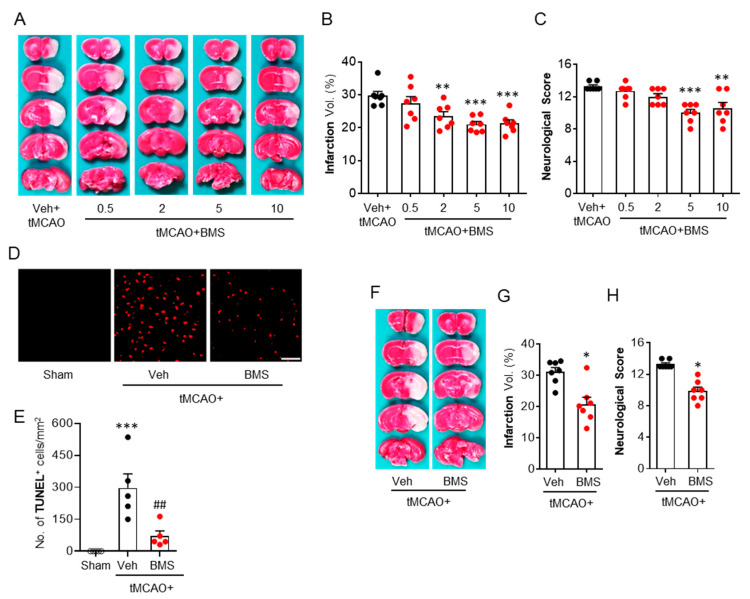
BMS administration reduces acute brain injuries in mice at one day after transient middle cerebral artery occlusion (tMCAO) challenge. Mice were challenged with tMCAO. BMS was administered to mice immediately after reperfusion (**A**–**E**) or at 3 h after reperfusion (**F**–**H**). Effects of BMS on brain damage were assessed at 24 h after tMCAO. (**A**–**C**) Effects of BMS at different dosages (0.5, 2, 5, and 10 mg/kg) on tMCAO-induced brain infarction (**A**,**B**) and neurological deficits (**C**). *n* = 7 mice per group. ** *p* < 0.01 and *** *p* < 0.001 versus vehicle-administered tMCAO mice (Veh + tMCAO). (**D**,**E**) Effects of BMS (5 mg/kg) on apoptotic cell death determined by TUNEL assay. Scale bar, 50 μm. *n* = 5 mice per group. *** *p* < 0.001 versus sham. ^##^ *p* < 0.01 versus vehicle-administered tMCAO mice (Veh + tMCAO). (**F**–**H**) Effects of delayed administration (at 3 h after reperfusion) of BMS on tMCAO-induced brain infarction (**F**,**G**) and neurological deficits (**H**). *n* = 7 mice per group. * *p* < 0.05 versus vehicle-administered tMCAO mice (Veh + tMCAO).

To determine the protective effects of BMS against acute brain injuries in mice after tMCAO challenge, brain infraction and neurological deficits in mice at 24 h after tMCAO challenge were assessed. We found that vehicle-administered mice showed increases in brain infarction in both the cortex and striatum, whereas BMS administration, dose-dependently, reduced brain infarct volume (Figure 1A,B). Similarly, tMCAO-induced neurological deficits were significantly attenuated by BMS administration, as evidenced by reduced neurological deficit scores (Figure 1C). Among different doses, BMS at 5 and 10 mg/kg attenuated brain infarction and neurological deficits the most, without showing any significant difference between these two doses (Figure 1A–C). Therefore, BMS at 5 mg/kg was used for further experiments in this study. BMS administration dramatically reduced numbers of TUNEL-positive cells in the ischemic core region compared to vehicle-administration control (Figure 1D,E), suggesting that BMS could reverse tMCAO-induced cell apoptosis in post-ischemic brains. Next, we investigated whether these neuroprotective effects of BMS could be preserved when BMS was administered at 3 h after reperfusion. This delayed administration of BMS also protected mice from tMCAO challenge as evidenced by reduced brain infarction (Figure 1F,G) and neurological deficit scores (Figure 1H). These data indicate that BMS administration can attenuate acute brain injuries in mice following ischemic stroke.

### 3.2. BMS Administration Attenuates tMCAO-Induced Microglial Activation and Lipid Peroxidation during the Acute Phase

**Figure 2:**
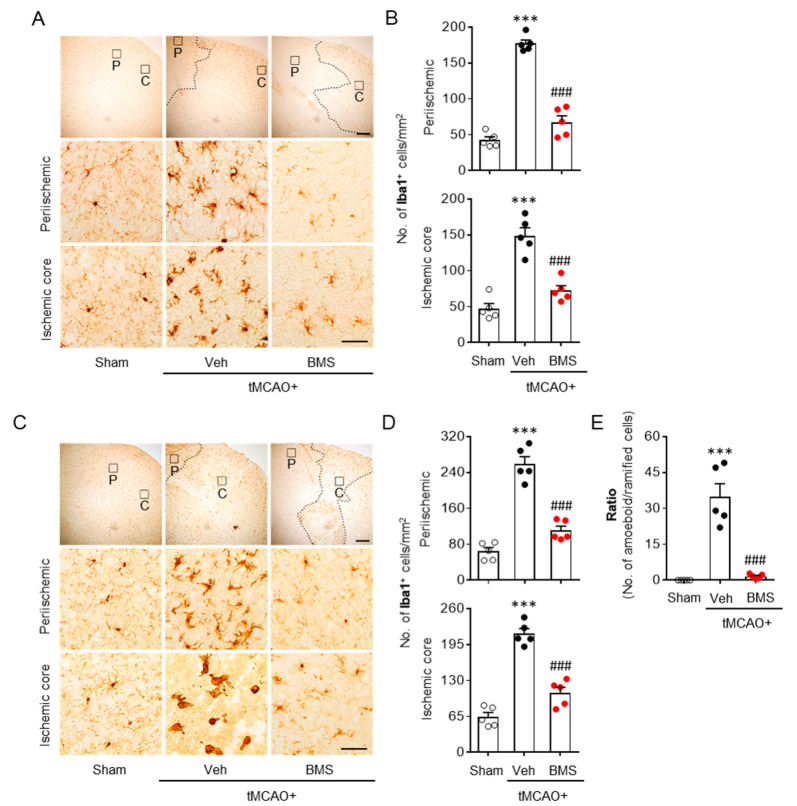
BMS administration attenuates microglial activation in mice at one day and three days after tMCAO challenge. Mice were challenged with tMCAO. BMS (5 mg/kg) was administered to mice immediately after reperfusion. Effects of BMS on tMCAO-induced microglial activation were assessed at one day (**A**,**B**) and three days (**C**–**E**) after tMCAO challenge. Representative images of Iba1-positive cells (**A**,**C**) in periischemic (P) and ischemic core (**C**) regions. Scale bars, 200 µm for top and 50 µm for middle and bottom. Quantification of Iba1-positive cells in periischemic and ischemic core (**B**,**D**) regions. (**E**) Quantification of ratio of amoeboid to ramified cells at three days after tMCAO challenge. *n* = 5 mice per group. *** *p* < 0.001 versus sham. ^###^ *p* < 0.001 versus vehicle-administered tMCAO mice (Veh + tMCAO).

The pathogenic role of LPA_1_ in an post-ischemic brain has been reported to be closely associated with microglial activation in periischemic and ischemic core regions in the acute phase (one and three days after tMCAO challenge) [12]. Therefore, we determined whether BMS administration could attenuate microglial activation in post-ischemic brains through Iba1 immunohistochemistry at 1 day and 3 days after tMCAO challenge. BMS administration dramatically attenuated tMCAO-induced microglial activation as evidenced by decreased numbers of Iba1-positive cells in periischemic and ischemic core regions at both 1 day (Figure 2A,B) and 3 days (Figure 2C,D) after tMCAO. It also dramatically attenuated the transformation of ramified microglia towards amoeboid microglia, more toxic forms of activated microglia, as shown by the attenuated ratio of amoeboid/ramified microglia at 3 days after ischemic challenge (Figure 2E). These results indicate that BMS administration can efficiently ameliorate tMCAO-induced microglial activation in injured brains during the acute phase.

**Figure 3:**
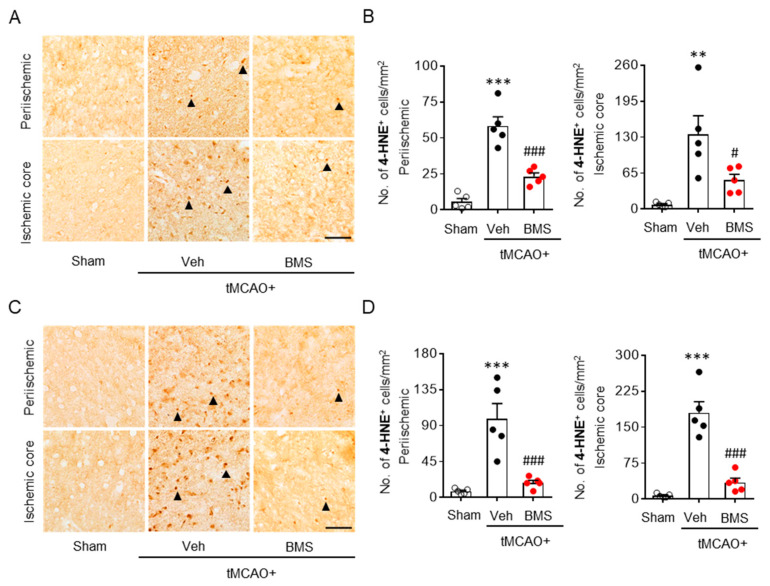
BMS administration attenuates lipid peroxidation in mice at one day and three days after tMCAO challenge. Mice were challenged with tMCAO. BMS (5 mg/kg) was administered to mice immediately after reperfusion. Effects of BMS on tMCAO-induced lipid peroxidation were assessed at one day (**A**,**B**) and three days (**C**,**D**) after tMCAO challenge. Representative images of 4-HNE-positive cells (**A**,**C**) in periischemic and ischemic core regions. Scale bars, 50 µm. Arrowheads indicate 4-HNE-positive cells. Quantification of 4-HNE-positive cells in periischemic and ischemic core regions (**B**,**D**). *n* = 5 mice per group. ** *p* < 0.01 and *** *p* < 0.001 versus sham. ^#^ *p* < 0.05 and ^###^ *p* < 0.001 versus vehicle-administered tMCAO mice (Veh + tMCAO).

Oxidative stress-mediated lipid peroxidation is a critical pathogenic event in post-ischemic brains. It mainly occurs during the acute phase of ischemic challenge [30]. LPA_1_ is associated with increased oxidative stress that induces retinal ganglionic cell degeneration [31]. Therefore, we determined whether BMS administration could attenuate lipid peroxidation in post-ischemic brains through 4-HNE immunohistochemistry at 1 day and 3 days after tMCAO challenge. BMS administration significantly decreased tMCAO-induced lipid peroxidation, as evidenced by reduced numbers of 4-HNE-positive cells in periischemic and ischemic core regions at both day 1 (Figure 3A,B) and day 3 (Figure 3C,D) after tMCAO challenge. These results indicate that BMS administration can also ameliorate tMCAO-induced oxidative stress in injured brains during the acute phase.

### 3.3. BMS Administration Attenuates tMCAO-Induced Neurological Deficits and Improves Survival Rate during the Sub-Acute Phase Along with Attenuation of Brain Tissue Loss and Cell Apoptosis

**Figure 4:**
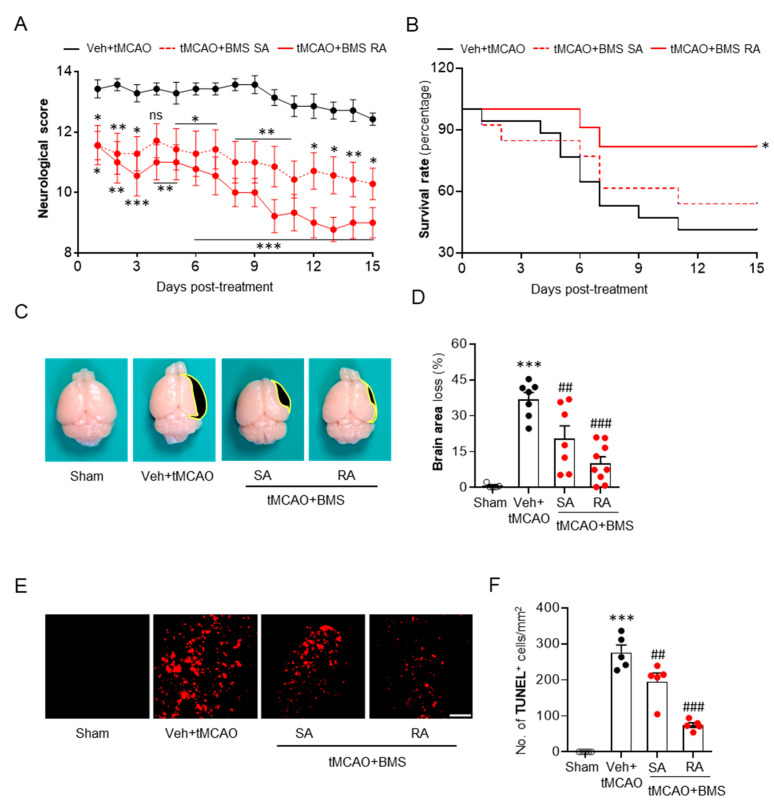
BMS administration reduces sub-acute brain injuries in mice for up to fifteen days after tMCAO challenge. Mice were challenged with tMCAO. BMS (5 mg/kg) was administered either once immediately after reperfusion (single administration of BMS, “SA”) or daily (repeated BMS administration, “RA”) for 14 days after tMCAO challenge. (**A**,**B**) Effects of BMS on neurological deficits (**A**) and survival (**B**) were determined for up to fifteen days after tMCAO challenge. *n* = 7 (Veh + tMCAO; black solid line), 7 (SA + tMCAO; red dotted line), and 9 (RA + tMCAO; red solid line). * *p* < 0.05, ** *p* < 0.01, and *** *p* < 0.001 versus vehicle-administered tMCAO mice (Veh + tMCAO). ns, not significant. (**C**,**D**) Effects of BMS on brain tissue loss were determined at day 15 after tMCAO. Representative brain images (**C**) and quantification (**D**) are shown. Black-colored areas in **C** indicate lost areas. *n* = 5 for sham, 7 for Veh + tMCAO, 7 for SA + tMCAO, and 9 for RA + tMCAO. *** *p* < 0.001 versus sham. ^##^ *p* < 0.01 and ^###^ *p* < 0.001 versus vehicle-administered tMCAO mice (Veh + tMCAO). (**E**,**F**) Effects of BMS on apoptotic cell death determined by TUNEL assay. Representative images of TUNEL-positive cells in the sub-ventricular zone (SVZ) (**E**) and quantification (**F**) are shown. Scale bar, 50 μm. *n* = 5 mice per group. *** *p* < 0.001 versus sham. ^##^ *p* < 0.01 and ^###^ *p* < 0.001 versus vehicle-administered tMCAO mice (Veh + tMCAO).

After confirming neuroprotective effects of BMS against acute brain injuries in tMCAO-induced mice, we next sought to determine whether these effects could be observed against sub-acute brain injuries following ischemic challenge by assessing its effects up to 15 days after tMCAO challenge. BMS was administered either once immediately after reperfusion (single administration) or daily for 14 consecutive days (repeated administration). Repeated BMS administration significantly lowered neurological deficit scores compared to vehicle administration control (Figure 4A). A single administration of BMS also attenuated neurological deficits, although the degree of its effectiveness was smaller than that by repeated administration (Figure 4A). In addition, repeated administration of BMS significantly increased the survival rate of tMCAO-challenged mice compared to the vehicle-administration control (Figure 4B). In the end point of the experiment (15 days after tMCAO), 41.2% of mice survived in the vehicle-administered group (Figure 4B). Single administration of BMS slightly but not significantly increased the survival rate to 53.8% compared to vehicle administration (Figure 4B). However, repeated administration of BMS significantly increased the survival rate to 81.8% (Figure 4B). We next determined whether BMS administration could attenuate tMCAO-induced brain atrophy. Either single or repeated administration of BMS significantly attenuated the tMCAO-induced brain tissue loss compared to vehicle administration, with more dramatic attenuation by repeated administration (Figure 4C,D). In addition, tMCAO-induced cell apoptosis was dramatically attenuated in the group with repeated administration of BMS, as evidenced by decreased numbers of TUNEL-positive cells (Figure 4E,F). In case of single administration of BMS, the number of TUNEL-positive cells was slightly but significantly reduced compared to that in the vehicle-administered group (Figure 4E,F). Taken together, these data clearly suggest that BMS administration can also exert neuroprotective effects against sub-acute brain injuries in mice following ischemic stroke.

### 3.4. BMS Administration Enhances Neurogenesis and Angiogenesis in Post-Ischemic Brains

**Figure 5:**
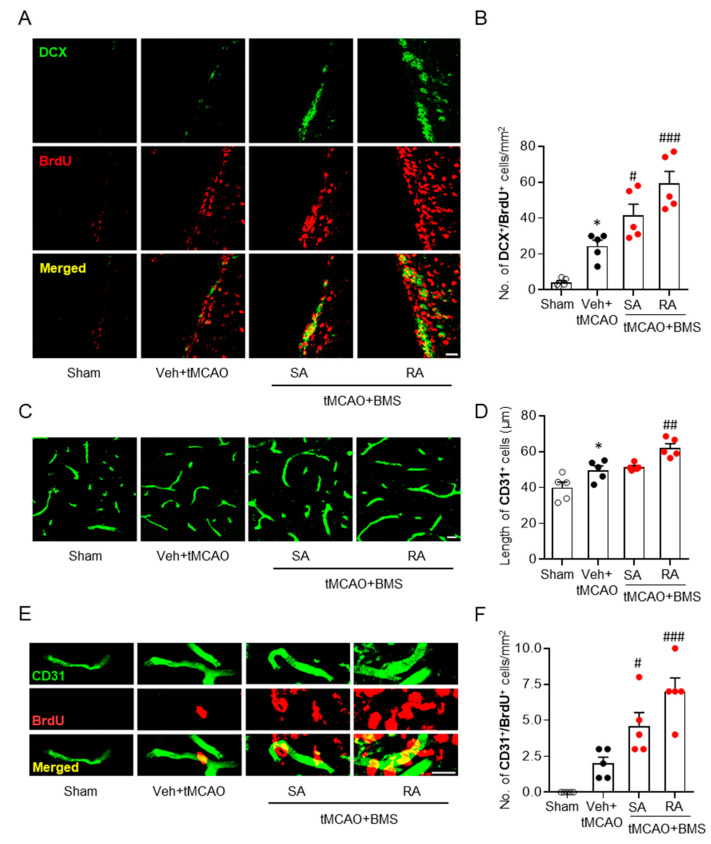
BMS administration promotes neurogenesis and angiogenesis in injured brains at fifteen days after tMCAO challenge. Mice were challenged with tMCAO. BMS (5 mg/kg) was administered either once immediately after reperfusion (single administration of BMS, “SA”) or daily (repeated BMS administration, “RA”) for 14 days after tMCAO challenge. (**A**,**B**) Effects of BMS on neurogenesis in the SVZ were determined by DCX/BrdU-double immunofluorescence staining. Representative images of DCX/BrdU-double positive cells in the SVZ (**A**) and quantification (**B**) are shown. (**C**–**F**) Effects of BMS on angiogenesis were determined by CD31/BrdU-double immunofluorescence staining. Representative images of CD31 (**C**) and quantification of lengths of vessels (**D**) are shown. Representative images of CD31/BrdU-double positive cells (**E**) and quantification of numbers of CD31/BrdU-double positive cells (**F**) are shown. Scale bars, 20 μm in A and E and 10 μm in C. *n* = 5 mice per group. * *p* < 0.05 versus sham. ^#^ *p* < 0.05, ^##^ *p* < 0.01, and ^###^ *p* < 0.001 versus vehicle-administered tMCAO mice (Veh + tMCAO).

Neurogenesis is an important neuroprotective event that can occur mainly in the sub-ventricular zone (SVZ) following tMCAO [32]. Newly born neurons can act as neuronal reservoirs to prevent disease pathogenesis in post-ischemic brains, at least in part [33]. Moreover, it is believed that potentiation of neurogenesis using exogenous intervention could be a desirable strategy for recovery after ischemic challenge [34,35]. To determine whether BMS administration could potentiate tMCAO-induced neurogenesis, we performed double immunofluorescence staining for newly born neurons with DCX and BrdU. Vehicle-administered tMCAO mice showed increased neurogenesis in the SVZ than sham-operated mice, as evidenced by increased numbers of BrdU/DCX-double positive cells (Figure 5A,B). BMS administration significantly increased neurogenesis than vehicle administration (Figure 5A,B). This enhancement was more obvious in the case of repeated administration of BMS than single administration (Figure 5A,B). These data suggest that BMS administration can enhance ischemic challenge-induced neurogenesis. This possibly contributes to its neuroprotective effects against sub-acute brain injuries following ischemic stroke.

Angiogenesis in post-ischemic brain, particularly in ischemic core regions, is another crucial mechanism for brain recovery after ischemic challenge [36,37]. To test whether long-term neuroprotective effects of BMS could also be associated with enhanced angiogenesis, BrdU/CD31 double immunofluorescence staining was performed. We found that lengths of CD31-positive vessels in ischemic core regions were significantly increased in the vehicle-administered group than in the sham group. Repeated administration of BMS significantly increased vessel lengths in ischemic core regions than vehicle administration (Figure 5C,D). Furthermore, it significantly increased numbers of CD31/BrdU-double positive cells in post-ischemic brains than vehicle administration (Figure 5E,F). In the group with single administration of BMS, numbers of CD31/BrdU-double positive cells (Figure 5E,F), but not lengths of CD31-positive vessels (Figure 5C,D), were significantly increased compared with those in the vehicle administration group, although these numbers were smaller than those in the group with repeated administration of BMS (Figure 5E,F). These data indicate that BMS administration can promote formation of new blood vessels in post-ischemic brains.

## 4. Discussion

The current study demonstrates that clinically-relevant BMS has neuroprotective potential against tMCAO-induced focal cerebral ischemia in mice. BMS administration protected mice from ischemic stroke during the acute phase (within one or three days) because it reduced tMCAO-induced brain infarction, neurological deficits, microglial activation, and lipid peroxidation. Importantly, BMS also showed promising neuroprotective effects during the sub-acute phase (up to fifteen days) because it attenuated neurological deficits and increased survival rate of tMCAO-challenged mice. These long-term neuroprotective effects of BMS were associated with enhanced neurogenesis and angiogenesis in post-ischemic brains. Findings of the current study strongly indicate a potential therapeutic benefit of BMS for ischemic stroke.

BMS is known to selectively antagonize LPA_1_. Previous reports have suggested that LPA_1_ signaling is involved in the pathogenesis of various neurological disorders including systemic sclerosis, neuropathic pain, spinal cord injury, hydrocephalus, hypoxia, and traumatic brain injury [13,15,16,17,38,39]. Indeed, our previous study has shown that inhibiting LPA_1_ activity by either a pharmacological antagonist or genetic deletion with a specific shRNA, can attenuate tMCAO-induced brain infarction and neurological deficits during the acute phase of ischemic stroke [12]. Such effects during the acute phase are associated with attenuation of microglial activation in post-ischemic brains [12]. Therefore, it is highly probable that BMS, a drug candidate for other diseases, can also exert certain neuroprotective effects against ischemic stroke-induced brain damage. Inevitably, BMS administration attenuated acute brain injuries such as brain infarction, functional neurological deficits, cell apoptosis, microglial activation, and lipid peroxidation following tMCAO challenge. Furthermore, an experiment for the therapeutic time window of BMS in the current study clearly showed that delayed administration of BMS could also effectively attenuate brain damage during the acute phase of ischemic stroke. BMS administration also dramatically attenuated oxidative stress in post-ischemic brains, as evidenced by reduced lipid peroxidation. Oxidative stress-induced ischemic brain damage is a critical pathogenic event in post-ischemic brains. Controlling oxidative damage could be a desirable therapeutic strategy for treating ischemic stroke [30]. In fact, LPA_1_ signaling is associated with modulation of oxidative damage in diverse disease conditions. LPA_1_ can trigger malondialdehyde release in radiation-induced pneumonia [40]. In addition, LPA_1_ is involved in oxidative stress-induced retinal ganglionic cell damage [31], indicating that LPA_1_ could influence oxidative brain damage in post-ischemic brains. In fact, the current study revealed that LPA_1_ activity in the ischemic brain could promote oxidative stress through increased lipid peroxidation for the first time. BMS dramatically attenuated ischemia-induced lipid peroxidation, suggesting that neuroprotective effects of BMS in acute ischemic stress might also be associated with attenuated oxidative stress in post-ischemic brains.

Long-term functional neurological impairment that leads to behavioral difficulties and disability is a major health concern associated with strokes [41]. Thus, it would be critical to address whether candidate agents could also be effective in protecting brains against injuries after ischemic challenge for a long time. Notably, the current study demonstrated that BMS could also provide long-term neuroprotection of mice from ischemic stroke. When BMS was given by either a single administration or repeated administration to tMCAO-challenged mice, attenuation of brain damage was observed for up to fifteen days (the end of experiments), indicating that BMS could be effective against sub-acute injuries after ischemic stroke. These data further indicate that inhibiting LPA_1_ activity can lead to long-term neuroprotection in ischemic stroke. Such pharmacological property of LPA_1_ inhibition was addressed for the first time in the current study. Apart from long-term neuroprotection by BMS, there seems to be a difference in the degree of effects between single administration and repeated administration of BMS. Although a single administration of BMS also attenuated neurological deficits, brain atrophy, and cell apoptosis during the sub-acute phase following ischemic challenge, its neuroprotective effects were less pronounced than repeated administration of BMS. The greater protective effect of repeated BMS administration than that of a single BMS administration was more evident in the case of survival of mice after ischemic stroke. In fact, a single administration of BMS failed to significantly improve the survival rate of those mice during the sub-acute phase, although the survival rate in the single administration group was higher than that in the vehicle-administered group. Considering that all determinations of sub-acute phase injuries—except survival rate—were performed for survived mice, overall neuroprotective effects by a single administration of BMS must be carefully interpreted. Nevertheless, our results still indicate that repeated BMS administration can provide significant long-term neuroprotection to mice from ischemic stroke.

Long-term neuroprotective effects of BMS appeared to be associated with enhanced neurogenesis in post-ischemic brains. Neurogenesis is one of crucial events involved in the long-term neuroprotection of ischemic brains [42]. Long-term disability in stroke survivors might be due to limited neurogenesis during post-stroke phases [43]. Therefore, therapeutic intervention that can enhance neurogenesis in post-ischemic brains may result in functional recovery [32,44]. In experimental rodent models of ischemic stroke, neurogenesis occurs mainly in the SVZ, starting from about the second week after tMCAO challenge [32]. Newly born neurons are believed to migrate towards injured sites to participate in ischemic recovery [42]. In the current study, it was evident that tMCAO caused neurogenesis in the SVZ. Such neurogenesis was further increased after BMS administration, suggesting that BMS-driven long-term neuroprotection might be due to such increased neurogenesis in an injured brain after ischemic stroke.

Angiogenesis is another crucial mechanism that can lead to long-term functional recovery and survival following a stroke [36,37], because angiogenesis can increase blood supply to the post-ischemic brain and prevent neuronal death. Therefore, promoting angiogenesis in post-ischemic brains could be another desirable strategy for developing therapeutics to manage secondary brain damage after a stroke incident [36]. In the current study, we found that repeated BMS administration increased numbers of newly-formed endothelial cells in injured brains after tMCAO challenge. Moreover, the length of vessels was significantly increased upon repeated BMS administration, indicating that BMS could promote tMCAO-induced angiogenesis. Therefore, the long-term neuroprotective potential of repeated BMS administration could be—at least in part—due to enhanced angiogenesis in post-ischemic brains. A single administration of BMS also exerted significant effects on numbers of newly-formed endothelial cells in injured brains after ischemic challenge, although it failed to exert statistically significant effects on the length of vessels compared to vehicle administration.

## 5. Conclusions

Taken together, our current study demonstrated that BMS administration could significantly improve ischemic stroke-induced brain damage and long-term disability. In particular, repeated BMS administration significantly induced neurogenesis and angiogenesis which might be underlying mechanisms responsible for the attenuated neurological deficits and higher survival rate caused by BMS administration. These aggregate results clearly suggest that clinically-relevant BMS might be a potential therapeutic agent for the management of stroke-induced long-term brain damage and functional disabilities. Moreover, targeting LPA_1_ could be a promising strategy to develop drugs for treating ischemic stroke.
